# SnO_2_ Anchored in S and N Co-Doped Carbon as the Anode for Long-Life Lithium-Ion Batteries

**DOI:** 10.3390/nano12040700

**Published:** 2022-02-19

**Authors:** Shuli Zhou, Hongyan Zhou, Yunpeng Zhang, Keke Zhu, Yanjun Zhai, Denghu Wei, Suyuan Zeng

**Affiliations:** School of Chemistry and Chemical Engineering, Liaocheng University, Liaocheng 252059, China; zhouli1996@126.com (S.Z.); zhy206533289@163.com (H.Z.); zyp2131@163.com (Y.Z.); zhukekexs@163.com (K.Z.); zhaiyanjun@lcu.edu.cn (Y.Z.); weidenghu@lcu.edu.cn (D.W.)

**Keywords:** S and N co-doped carbon, capacity contribution, cyclic stability, lithium-ion batteries

## Abstract

Tin dioxide (SnO_2_) has been the focus of attention in recent years owing to its high theoretical capacity (1494 mAh g^−1^). However, the application of SnO_2_ has been greatly restricted because of the huge volume change during charge/discharge process and poor electrical conductivity. In this paper, a composite material composed of SnO_2_ and S, N co-doped carbon (SnO_2_@SNC) was prepared by a simple solid-state reaction. The as-prepared SnO_2_@SNC composite structures show enhanced lithium storage capacity as compared to pristine SnO_2_. Even after cycling for 1000 times, the as-synthesized SnO_2_@SNC can still deliver a discharge capacity of 600 mAh g^−1^ (current density: 2 A g^−1^). The improved electrochemical performance could be attributed to the enhanced electric conductivity of the electrode. The introduction of carbon could effectively improve the reversibility of the reaction, which will suppress the capacity fading resulting from the conversion process.

## 1. Introduction

Lithium-ion batteries (LIBs) have been the focus of attention nowadays because of their advantages, such as their long lifetime, no memory effect, high energy density and their light weight [1,2,3,4]. However, the low theoretical value (372 mAh g^−1^) as well as poor cycling performance of graphite cannot meet the ever increasing requirement nowadays, especially in the fields of electrical vehicles [5,6,7]. Thus, exploring new material systems with higher capacity, long cycling life and lower cost are in urgent need.

Tin dioxide (SnO_2_) has been considered to be an ideal candidate as the anode material in LIBs because of its high theoretical specific capacity (up to 1494 mAh g^−1^) based on both conversion and alloying reaction mechanism [8,9,10,11]. However, the huge volume change (>300%) during the alloying/de-alloying process will lead to the pulverization of SnO_2_ and result in the rapid capacity fading during the charge/discharge process. Meanwhile, the poor electrical conductivity of SnO_2_ will also aggravate the pulverization process, which further worsens the capacity fading. To improve the electrochemical performance of SnO_2_, the key point is to enhance the structural stability of SnO_2_. Nowadays, this aim is mainly realized via two methods. The first method is based on the morphological control of SnO_2_, which result in the fabrication of SnO_2_ with different morphologies during the past years [12,13,14,15,16,17,18]. While the second method is based on the incorporation of carbon with SnO_2_. The introduction of carbon will increase the electrical conductivity of the electrode material as well as buffer the volumetric expansion during the charge/discharge process, both of which are beneficial for the improvement of the electrochemical performances. For example, Guan et al. have encapsulated porous SnO_2_ into carbon, and the as-obtained G@p-SnO_2_@C composite can deliver a specific capacity of 417 mAh g^−1^ even after 1800 cycles at a high current density of 1.5 A g^−1^, which exhibits excellent cycling stability owing to the existence of porous carbon [19]. Xu et al. have reported the synthesis of microbelt–void–microbelt structured SnO_2_@C composite [20]. The void space between the carbon shell and SnO_2_ microbelt could perfectly relieve the volumetric expansion during the alloying process. As a result, the as-prepared composite structures exhibit excellent cycling stability. Even after 300 charge/discharge cycles under the current density of 0.3 A g^−1^, a discharge capacity of 1227 mAh g^−1^ can still reach. Kamali’s group decorated SnO_2_ nanorods with natural graphite (NG-SnO_2_) through a molten salt method. Additionally, the hybrid material delivered an excellent electrochemical performance (capacity: 495 mAh g^−1^ after 500 cycles) [21]. Ha et al. deposited SnO_2_ nanoparticles on the carbon nanofibers (CNF) and prepared SnO_2_@CNF composites by a hydrothermal method. Compared to pristine SnO_2_, the best SnO_2_@CNF composites shows an improved specific capacity (909 mAh g^−1^) at 0.1 A g^−1^ after 100 cycles [22]. All these experimental facts clearly indicate that the combination of SnO_2_ with carbon is an effective method to improve the electrochemical performance of SnO_2_. However, these synthetic methods are usually too complicated, which prevent it from large-scale application in lithium-ion battery. A simple and facile method for the synthesis of SnO_2_@C composite materials is still in urgent need.

Compared to pristine carbon, the heteroatom doping of (S, N and P) carbon can further enhance the electrical conductivity of carbon, which will render it fast diffusion of active metal ions [23,24,25,26,27,28]. Therefore, combining heteroatom-doped carbon with SnO_2_ could promote the electrochemical performance further, which is seldom reported in the previous reports. Herein, a simple solid-state reaction was employed for the synthesis of SnO_2_@SNC composite (SNC: S and N co-doped carbon) structures. After the introduction of carbon, the electrochemical properties of the composite materials are greatly enhanced. Compared with pristine SnO_2_, the as-prepared SnO_2_@SNC composite materials exhibit excellent cycling performance. Even after cycling for 1000 times, the as-synthesized SnO_2_@SNC can still deliver a discharge capacity of 600 mAh g^−1^ (current density: 2 A g^−1^). Further experiments suggest that the decrease in the resistance of the electrode is the key point for the cycling stability of the as-prepared samples. By lowering the electrical resistance of the electrode, the reversibility of the reaction is greatly enhanced, leading to the excellent cycling performance of the samples.

## 2. Materials and Methods

### 2.1. Chemicals

All chemicals were of analytical grade and used without further purification, including chlorotriphenyltin (C_18_H_15_ClSn), L-cysteine (C_3_H_7_NO_2_S) and sodium chloride (NaCl).

### 2.2. Synthesis of SnO_2_@SNC Composite Structures

**Figure 1:**
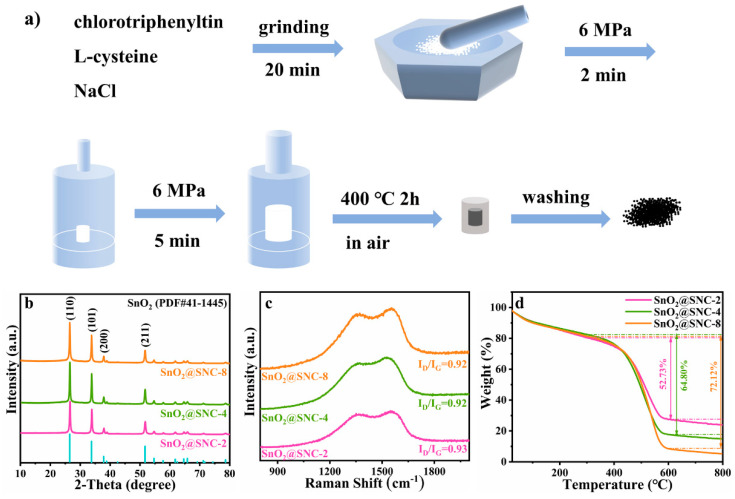
(**a**) Schematic illustration for the synthetic process of the SnO_2_@SNC composite materials. (**b**) XRD pattens, (**c**) Raman spectra and (**d**) TGA curves for the as-prepared SnO_2_@SNC−2, SnO_2_@SNC−4 and SnO_2_@SNC−8 composite.

The SnO_2_@SNC composites were obtained by a simple solid-state reaction. Additinally, the schematic illustration of the synthetic process is shown as Figure 1a. In a typical process, 2 mmol (0.7709 g) of chlorotriphenyltin (C_18_H_15_ClSn), 4 mmol (0.4846 g)/8 mmol (0.9692 g)/16 mmol (1.9384 g) of L-cysteine (C_3_H_7_NO_2_S) and 1.2555/1.7401/2.7093 g of sodium chloride (NaCl) were ground together in an agate mortar for 20 min. The mixture was then transferred into a stainless-steel mold with diameter of 10 mm and pressed into a small column (6 MPa for 2 min). In the next step, the as-formed column was encapsulated by a NaCl layer and press into a larger column with diameter of 20 mm in another mold (6 MPa for 5 min). Then, the as-formed column was calcined at 400 °C for 2 h in a muffle furnace (heating rate: 2 °C min^−1^). After being cooled down to room temperature, the column was put into the water to remove the excessive NaCl. The final product was obtained by centrifugation accompanied by washing with de-ionized water and ethanol three times. After being dried at 60 °C for 12 h under vacuum, black SnO_2_@SNC composite was obtained. The three samples that synthesized with different amount of L-cysteine (molar ratios between chlorotriphenyltin and L-cysteine are 1:2/1:4/1:8) were labeled as SnO_2_@SNC-2, SnO_2_@SNC-4 and SnO_2_@SNC-8, respectively. For comparison purposes, pure SnO_2_ without carbon was also synthesized using the same procedure except for the addition of L-cysteine. To illustrate the effect of S and N doping, SnO_2_@C without S and N doping was also synthesized by replacing L-cysteine with starch, keeping the other reaction conditions constant.

### 2.3. Materials Characterization

X-ray power diffraction (XRD) patterns of the SnO_2_@SNC samples were recorded on a diffractometer (Rigaku Smartlab 9, Tokyo, Japan) with Cu Kα radiation during a scan range of 10–80° at a scan rate of 20°/min. Raman spectra of the samples were measured by an Invia Raman microscope (λ = 532 nm)(Invia Microscope, Renishaw, Wotton-under-Edge, Gloucestershire, UK). X-ray photoelectron spectrum (XPS) were obtained by ESCALAB 250 instrument (Thermo Fischer, Waltham, MA, USA) to investigate the chemical states and compositions. The morphologies of the samples were examined by a scanning electron microscopy (SEM, Thermo Fisher, Helios CX, Waltham, MA, USA) and high-resolution transmission electron microscopy (HRTEM, Thermo Fisher, Talos F200x, Waltham, MA, USA). The BET surface areas as well as size distributions for the samples were measured using the N_2_ adsorption–desorption instrument (Micromeritics ASAP 2460, Missouri, USA). The thermogravimetric analysis was conducted using a thermal gravimetric analyzer (NETZSCH STA F5, Selb, Germany, mass loading: 10.2 mg, heating rate: 10 °C min^−1^, atmosphere: air).

### 2.4. Electrochemical Measurement

The electrochemical measurements were tested by CR2032 coin cell using lithium foil as the counter electrode and Celgard 2400 membrane as the separator. The working electrode was prepared using the slurry composing of active materials, carbon black (Super P) and sodium carboxymethyl cellulose (CMC) binder (7:2:1, wt%). Then, the slurry was coated on copper foil and was dried in a vacuum oven at 100 °C for 12 h. The mass loading on copper foil was around 1.0 mg/cm^2^. The electrolyte was LiPF_6_ dissolving in a mixture of ethylene carbonate (EC) and diethyl carbonate (DEC) (1:1 vol%). The CR2032 coin cell was assembled in the glovebox filled with argon atmosphere (H_2_O < 0.01 ppm, O_2_ < 0.01 ppm). The galvanostatic charge and discharge process was measured on LAND CT2001A system at a range of 0.01 V and 3 V. Additionally, the tested batteries were activated at a current density of 0.1 A g^−1^ for the first three cycles. Rate performances under different current densities were tested using the same instrument. Cyclic voltammetry (CV, 0.01–3.0 V) and electrochemical impedance spectroscopy (EIS, 0.01–10^5^ Hz) were measured using the Gamry electrochemical workstation. All assembled batteries were set still for at least 12 h at room temperature before testing.

## 3. Results and Discussions

### 3.1. Composition and Microstructures of SnO_2_@SNC Composite Materials

The SnO_2_@SNC composites were synthesized by the so-called bake-in-salt method (Figure 1a) which has been reported for the synthesis of Mn_3_O_4_@C composite material in our previous work [29]. During the synthetic process, NaCl will act as both template and heat-conducting medium. Upon heating, both chlorotriphenyltin (melting point: 108 °C) and L-cysteine (melting point: 240 °C) will melt and mix together in the first step. Then, the liquid phase mixture will diffuse into the micro-channels between the NaCl particles because of the caterpillar force, which will lead to the formation of the mesoporous structures. Upon further heating, both the chlorotriphenyltin and L-cysteine will decompose, resulting in the formation of SnO_2_@SNC composite structures. Because of the chemical composition of L-cysteine, the S, N co-doped carbon will form during the decomposition process. Figure 1b shows the XRD patterns of the as-synthesized three SnO_2_@SNC materials, and all the diffraction peaks on which can be indexed to be a tetragonal phased SnO_2_ (JCPDS Card No. 41-1445). The peaks centering at 26.61, 33.89, 37.95 and 51.78° can be assigned to the (110), (101), (200) and (211) crystal planes of tin dioxide (SnO_2_), respectively. However, the diffraction peak corresponding to carbon was not observed on the XRD patterns, which may result from the amorphous nature of carbon in the three samples. To verify the existence of carbon in the final products, Raman spectroscopy was employed. The Raman spectra of the three as-prepared SnO_2_@SNC samples were shown in Figure 1c, which clearly indicate the existence of carbon in the final products. Two broad peaks centering at 1364 and 1555 cm^−1^ can be observed, which can be ascribed to the lattice defect of carbon (D band) and the in-plane stretching vibrations of C sp^2^ hybridization (G band). The value of I_D_/I_G_ for the as-prepared three samples are determined to be 0.92, 0.92 and 0.93, illustrating the high graphitization degree for the 3 SnO_2_@SNC samples [1,30]. To determine the carbon contents for the three SnO_2_@SNC samples, thermogravimetric analysis (TGA) was employed (Figure 1d). Before 300 °C, the weight loss of the samples can be ascribed to the loss of the adsorbed water or other small molecules. From 300 to 600 °C, the sharp weight loss is related to the combustion reaction of amorphous carbon. The carbon contents for the SnO_2_@SNC-2, SnO_2_@SNC-4 and SnO_2_@SNC-8 were determined to be 52.73%, 64.80% and 72.12%, respectively, which clearly indicate that the carbon contents of the final products can be effectively adjusted by controlling the initial amount of L-cysteine in the starting material. For comparison purpose, the XRD pattern, Raman spectra and TGA curve of pristine SnO_2_ are also recorded and shown in Figure S1.

**Figure 2:**
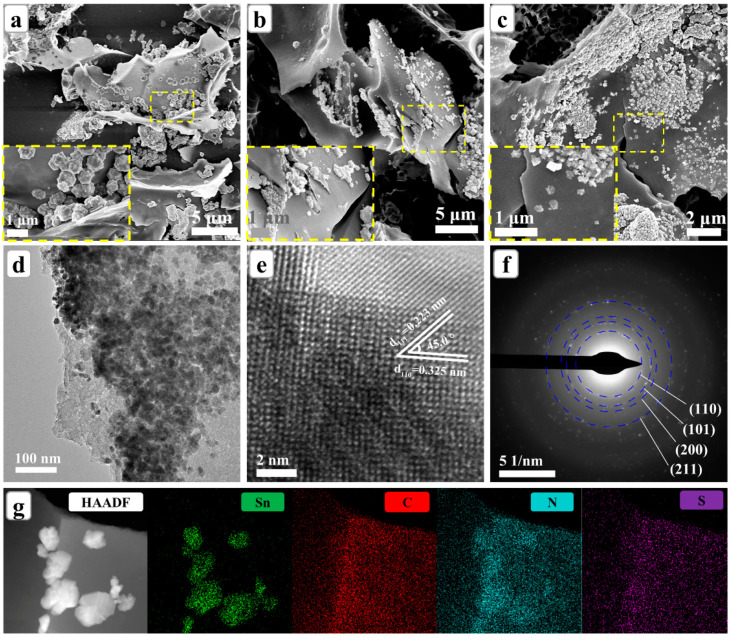
SEM images for the (**a**) SnO_2_@SNC−2, (**b**) SnO_2_@SNC−4 and (**c**) SnO_2_@SNC−8 composite. (**d**) TEM image, (**e**) HRTEM and (**f**) SAED spectra of the SnO_2_@SNC−8 sample. (**g**) HAADF image and elemental mapping for Sn, C, N and S element of SnO_2_@SNC−8 composite.

The structural as well as morphological features of the three samples were investigated using the field-emission scanning electron microscopy (FE-SEM). Figure 2a–c are the SEM images for samples SnO_2_@SNC-2, SnO_2_@SNC-4 and SnO_2_@SNC-8 with different magnifications. The experimental facts clearly indicate the distribution of SnO_2_ particles on the surfaces of carbon. Based on these SEM images results, the sizes of SnO_2_ particles were approximately 650, 400 and 150 nm for SnO_2_@SNC-2, SnO_2_@SNC-4 and SnO_2_@SNC-8 composites. The corresponding TEM image in Figure 2d demonstrates that SnO_2_ particles anchored on the carbon matrix. The high-resolution transmission electron microscopy (HRTEM) image (Figure 2e) shows clear lattice fringe with an inter-planar spacing of 0.325 and 0.223 nm, which is consistent to the d-spacings of the (110) and (111) lattice planes of SnO_2_, respectively. As shown in Figure 2f, the selected area electron diffraction (SAED) pattern for sample SnO_2_@SNC-8 clearly demonstrates the poly-crystalline natures of the SnO_2_ nanoparticles. The four ring-like diffraction patterns can be perfectly indexed to (110), (101), (200) and (211) lattice plane of tetragonal phase SnO_2_, which is consistent with the HRTEM observation. To further understand the elemental distribution of the SnO_2_@SNC-8 composite, the elemental mapping was carried out. The experimental facts clearly demonstrate the distribution of SnO_2_ nanoparticles on the carbon nanoplates (Figure 2g). Furthermore, the well-distributed S, N and C elements in the plate also indicates that S and N are successfully doped in the carbon matrix. The dispersed SnO_2_ nanoparticles and the incorporation of the carbon matrix may contribute to enhance the cycling stability of the composite. The void space among the carbon nanosheets is beneficial to the penetration and surface contact of the electrolyte, which will effectively improve the structure stability during the cycling process. For pristine SnO_2_, only SnO_2_ nanoparticles are observed in the sample (Figure S2).

**Figure 3:**
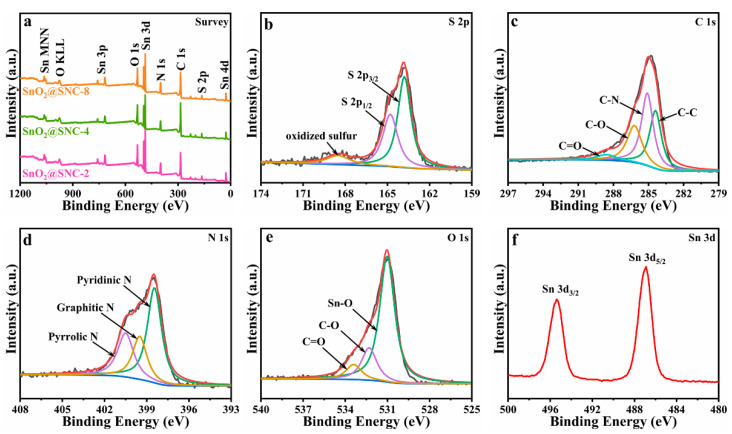
(**a**) XPS survey for SnO_2_@SNC−2, SnO_2_@SNC−4 and SnO_2_@SNC−8 sample. (**b**) S 2p, (**c**) C 1s, (**d**) N 1s, (**e**) O 1s and (**f**) Sn 3d high resolution XPS spectrum for SnO_2_@SNC−8 composite.

To get further insight into the chemical states and compositions of the samples, X-ray photoelectron spectroscopy (XPS) analysis was employed. The overall survey spectra clearly indicate the existence of elements Sn, O, S, N and C for the three samples (Figure 3a). Figure 3b is the detailed XPS spectrum of S, where the three peaks centering at 163.9, 164.8 and 168.5 eV can be ascribed to S 2p_3/2_, S 2p_1/2_ and the oxidized sulfur, respectively [31]. The C 1s high resolution spectrum (Figure 3c) can be divided into four peaks corresponding to C–C (284.34 eV), C–N (285.07 eV), C–O (286.27 eV) and C=O (288.65 eV) bond, respectively [11]. The presence of C–N chemical bond clearly indicates that doping of element N in the carbon matrix. The high-resolution spectrum of N 1s is shown in Figure 3d, where binding energies of 398.4, 399.5 and 400.5 eV can be attributed to pyridinic N, graphitic N and pyrrolic N, respectively [32]. As it is shown in Figure 3e, the 3 peaks centering at 531.1, 532.3 and 533.4 eV can be ascribed to Sn–O, C=O and C–O chemical bond, respectively [33]. Figure 3f is the XPS spectrum of the Sn 3d peak, on which the 2 peaks centering at 495.3 and 486.9 eV can be ascribed to Sn 3d_3/2_ and Sn 3d_5/2_, respectively. The result clearly indicates the presence of Sn^4+^ in the SnO_2_@SNC-8 composite material [34]. The difference value between the two peaks is determined to be 8.4 eV, which is consistent with the previous reports for pristine SnO_2_ [35,36,37].

**Figure 4:**
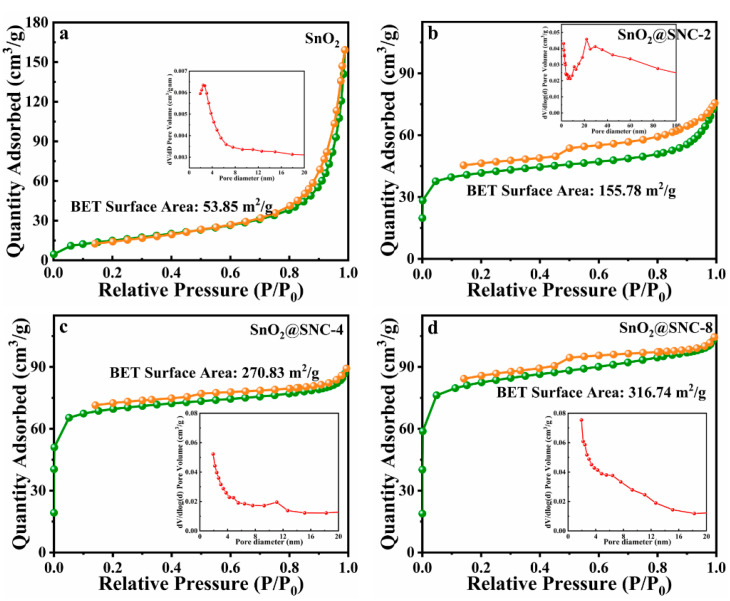
N_2_ adsorption-desorption isotherm (the insets showing the pore size distribution) for (**a**) pure SnO_2_, (**b**) SnO_2_@SNC−2, (**c**) SnO_2_@SNC−4 and (**d**) SnO_2_@SNC−8 composite.

The N_2_ adsorption-desorption isotherms and size distributions for all the samples were also measured (Figure 4). The Brunauer–Emmett–Teller (BET) surface areas of the four samples are determined to be 53.85, 155.78, 270.83 and 316.74 m^2^/g, respectively. Obviously, the incorporation of SnO_2_ with the S, N co-doped carbon effectively increase the surface areas of the samples. Upon the increase in carbon contents, the surface areas of the samples gradually increase.

### 3.2. Electrochemical Property in Half-Cells

**Figure 5:**
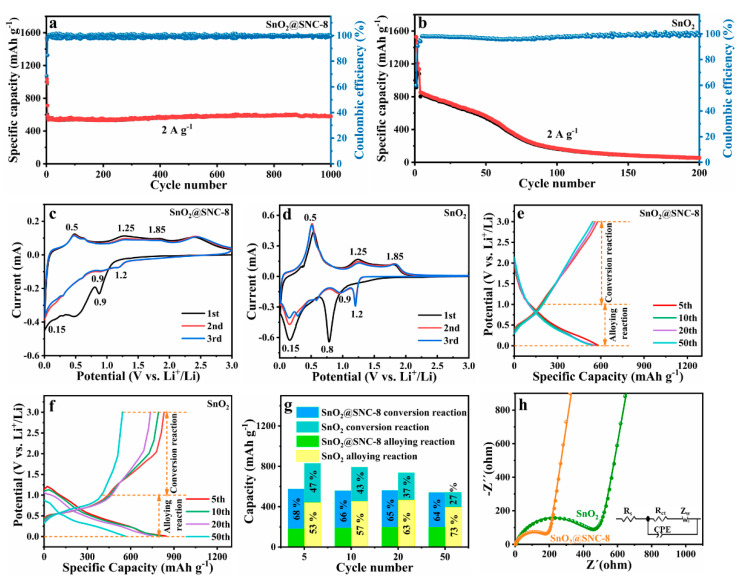
Long cyclic performance at 2 A g^−1^ for the (**a**) SnO_2_@SNC−8 and (**b**) pure SnO_2_. CV curves at a scan rate of 0.1 mV s^−1^ in the first three cycles for the (**c**) SnO_2_@SNC−8 and (**d**) pure SnO_2_. Galvanostatic charge/discharge voltage profiles at 2 A g^−1^ for the (**e**) SnO_2_@SNC−8 and (**f**) pure SnO_2_. (**g**) The charge capacity from the process of conversion reaction and alloyed reaction at the 5th, 10th, 20th and 50th cycle for SnO_2_@SNC−8 and SnO_2_. (**h**) EIS comparison for SnO_2_@SNC−8 and SnO_2_ sample and the corresponding equivalent circuit model.

To evaluate the electrochemical performances of the as-prepared SnO_2_@SNC composite materials, the long-life cycle performances of the samples were tested within a voltage of 0.01–3.0 V (current densities: 2 A g^−1^). Obviously, the as-obtained sample SnO_2_@SNC-8 exhibits excellent cycling stability during the charge–discharge process (Figure 5a). Even after cycling for 1000 times under the current density of 2 A g^−1^, the as-prepared sample SnO_2_@SNC-8 can still deliver a discharge capacity of ~600 mAh g^−1^. To understand the effect of S and N doping, the electrochemical properties of sample SnO_2_@C (Figure S3) were also investigated. As it is shown in Figure S4, an obvious capacity fading can be observed during the cycling process. After cycling 130 times under the current density of 2 A g^−1^, the as-prepared sample SnO_2_@C can only deliver a discharge capacity of ~300 mAh g^−1^. In the next step, the long-life cycling performance of pristine SnO_2_ was also evaluated (Figure 5b). The discharge capacity of the pristine SnO_2_ rapidly decreased to ~55 mAh g^−1^ after 200 cycles under the same conditions. The experimental results clearly indicate that the introduction of carbon can obviously improve the cycling stability of the sample. According to the previous reports, the serious capacity fading for the SnO_2_ materials can be mainly ascribed to the irreversible transition reaction and huge volume change during alloying reaction [38,39,40,41]. The introduction of carbon will buffer the volume expansion during the charge/discharge process, which will be beneficial to the cycling performances of the sample. After S and N doping, the electrochemical performance of SnO_2_ can be further improved. As a result of S- and N-doped carbon, the as-prepared SnO_2_@SNC-8 exhibits excellent cycling stability. Compared with the original carbon, doped carbon materials with heteroatoms (N, S) could further facilitate the diffusion of active metal ions, rendering the enhanced conductivity of carbon, thereby accelerating the electron transport of carbonaceous materials. As a result, the as-prepared sample SnO_2_@SNC-8 exhibits excellent electrochemical properties.

To further reveal the role that carbon played during the charge/discharge process, a series of characterizations were employed. In the first step, the electrochemical reaction processes for samples SnO_2_@SNC-8 and SnO_2_ were evaluated by CV with a scan rate of 0.1 mV s^−1^ (Figure 5c,d). For the two samples, the curves in the first cathodic scan are different to the second and third cycles, resulting from the formation of solid electrolyte interfaces (SEI) film on the surfaces of active materials and irreversible electrolyte decomposition. For the second and third cathodic scan processes, the peaks centering at 1.2 and 0.9 V can be ascribed to the reduction process from SnO_2_ to Sn (SnO_2_ + 4 Li^+^ + 4 e^−^ → Sn + 2 Li_2_O). The peak centering at ~0.15 V can be ascribed to the alloying reaction from Sn to Li–Sn alloy. In the anodic scan process, the peak centering around 0.5 V corresponds to the de-alloy process of Li–Sn alloy. The broad peak centering at ~1.25 and ~1.85 V is related to oxidation process from Sn to SnO_2_. Figure S5a,b are the galvanostatic charge/discharge voltage profiles of SnO_2_@SNC-8 and pure SnO_2_ for the first three cycles (current density: 100 mA g^−1^), the initial coulombic efficiency (ICE) of sample SnO_2_@SNC-8 is measured to be 68.64%, which is higher than pristine SnO_2_ (58.88%). The initial irreversible capacity loss is usually related to the SEI film formation and side reaction during the charge/discharge process [39,42,43,44], and incorporation of carbon with SnO_2_ can improve the ICE value.

SnO_2_ + 4 Li^+^ + 4 e^−^ ↔ Sn + 2 Li_2_O (1)

Sn + x Li^+^ + x e^−^ ↔ Li_x_Sn (0 ≤ x ≤ 4.4) (2)

According to the CV results of the two samples, both the redox process and the alloying/de-alloying process play important roles for the capacities of the two samples (Equations (1) and (2)). To figure out the exact role of carbon during the two processes, the capacity contribution of the two processes is calculated according to the galvanostatic charge/discharge curves (Figure 5e,f). Obviously, the GCD curves for SnO_2_@SNC-8 composite nearly overlap, indicating good reversibility during the charge/discharge process (Figure 5e). Combining the CV curves and GCD results, we can draw the conclusion that the de-alloy process mainly occurred when the voltage is below 1.0 V. While for the conversion reaction, it mainly happens when the voltage is above 1.0 V during charge process [37]. Thus, the capacity can be divided into two parts according to the different reaction mechanism and the corresponding results are shown as Figure 5e,f. For the alloy reaction part (below 1.0 V), the specific capacities of SnO_2_@SNC-8 at 5th, 10th, 20th and 50th cycle were calculated to be 182, 190, 198 and 198 mAh g^−1^. While for pure SnO_2_, the corresponding values were determined to be 442, 455, 466 and 396 mAh g^−1^ (Figure 5g), indicating unchanged values for both SnO_2_@SNC-8 and pure SnO_2_. For the conversion reaction part (above 1.0 V), the specific capacities of SnO_2_@SNC-8 at 5th, 10th, 20th and 50th cycle were calculated to be 395, 370, 364 and 345 mAh g^−1^. While for pure SnO_2_, the corresponding values were determined to be 389, 338, 271 and 138 mAh g^−1^, exhibiting a sharp decrease in specific capacity. The experimental facts clearly indicate that the capacity fading for pure SnO_2_ mainly result from the irreversible conversion reaction during the cycling process. As it is known, the pulverization of SnO_2_ particles during cycling is mainly related to the intrinsic low electric conductivity of SnO_2_, which will lead to the irreversible transitional reaction and result in the capacity fading [45]. The combination of SnO_2_ with carbon can reduce the electrical resistance and effectively improve the reversibility of the conversion reaction. To verify this viewpoint, electrochemical impedance spectroscopy (EIS) was carried out (Figure 5h). According to the equivalent circuit (Figure 5h inset), the fitted resistance values are shown in Table 1. The ohmic resistance (R_s_) corresponds to the interception of Z’, which includes the sum of the electrolyte, separator and contact resistance. The charge transfer resistance (R_ct_) represents resistance at the electrode/electrolyte interfaces and Warburg resistance (Z_w)_ relates to lithium diffusion rate. Apparently, the SnO_2_@SNC-8 exhibited lower R_s_ (2.286 Ω), R_ct_ (166.9 Ω) and Z_w_ (127.3 Ω s^−1/2^) values than pristine SnO_2_ (6.569 (R_s_), 391.5 (R_ct_) and 320.2 Ω (Z_w_ s^−1/2^)), indicating the introduction of S and N co-doped carbon could enhance the electrical conductivity and is beneficial to accelerate charge transfer and lithium diffusion.

**Figure 6:**
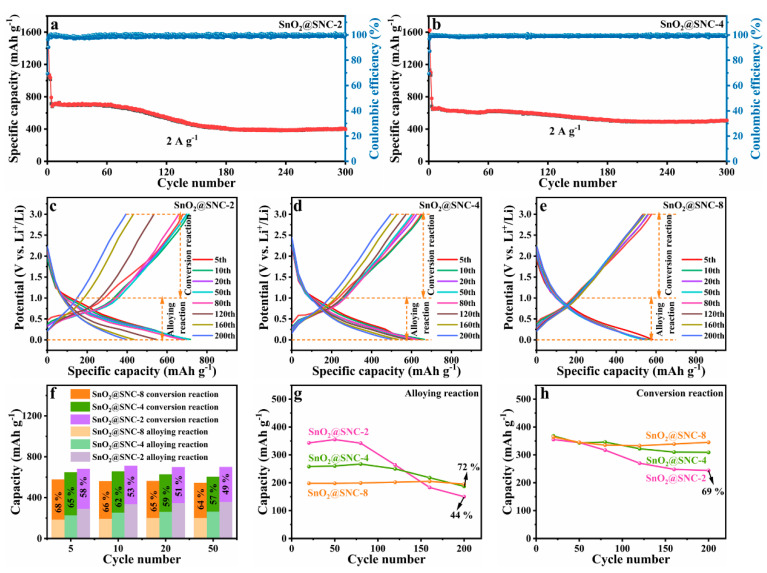
Long cyclic performance at 2 A g^−1^ for the (**a**) SnO_2_@SNC−2 and (**b**) SnO_2_@SNC−4. GCD profiles at 2 A g^−1^ for the (**c**) SnO_2_@SNC−2, (**d**) SnO_2_@SNC−4 and (**e**) SnO_2_@SNC−8 at the 5th, 10th, 20th, 50th, 80th, 120th, 160th and 200th cycle. (**f**) The capacity contribution of SnO_2_@SNC−2, SnO_2_@SNC−4 and SnO_2_@SNC−8 from the process of conversion reaction and alloyed reaction at the 5th, 10th, 20th and 50th cycle. The capacity retention of (**g**) alloying reaction and (**h**) conversion reaction at 2 A g^−1^ for the three samples.

To get further insight into the effects of carbon, the electrochemical performances of samples with different carbon contents were also investigated. Although, SnO_2_@SNC-2 and SnO_2_@SNC-4 have a similar electrochemical mechanism with SnO_2_@SNC-8; according to the CV profiles (Figure S6), obvious differences in long-life performances can still be observed (Figure 6a,b). For sample SnO_2_@SNC-2, an obvious capacity fading can be clearly observed after charging/discharging for 80 cycles (Figure 6a). While for sample SnO_2_@SNC-4, the capacity starts to decreases from 80 cycles until 200 cycles and then increases slowly in the following cycles (Figure 6b). Obviously, SnO_2_@SNC-8 exhibits the best long-life performance as compared to SnO_2_@SNC-2 and SnO_2_@SNC-4 under the same current density. The capacity contribution from different reaction process at 2 A g^−1^ in the 5th, 10th, 20th and 50th cycle is also calculated (Figure 6c–f). As it is shown in Figure 6f, the SnO_2_@SNC-2 and SnO_2_@SNC-4 have a similar reaction ratio with SnO_2_@SNC-8, indicating the positive effect of carbon for the maintaining of the reaction reversibility during the charge/discharge process. However, an obvious capacity fading between 80 and 200 cycles was observed for samples SnO_2_@SNC-2 and SnO_2_@SNC-4, which is not observed for SnO_2_@SNC-8. To study the capacity fading mechanism, the capacity contribution during the first 200 cycles were calculated and the corresponding results are plotted (Figure 6g–h). For sample SnO_2_@SNC-8, the specific capacity resulting from both alloying and conversation reaction was maintained as stable during the cycling process, which is consistent with long-life performance. For sample SnO_2_@SNC-4, the conversion process remains unchanged during the cycling process. However, the capacity resulting from the alloying process decreases by 28%, indicating the capacity fading of SnO_2_@SNC-4 mainly result from the irreversibility of the alloy reaction. For sample SnO_2_@SNC-2, capacities resulting from both the alloy process (56%) and the conversion process (31%) decreases, leading to the capacity fading during the cycling process. Obviously, the increase in carbon content greatly enhanced the reversibility of the reaction process. Furtherly, as shown in Figure S7a, we also tested the rate performance at 0.2, 0.5, 1.0, 2.0, 5.0 and 10.0 A g^−1^, respectively. Compared to samples SnO_2_@SNC-2 and SnO_2_@SNC-4, sample SnO_2_@SNC-8 exhibits the best rate performance. For sample SnO_2_@SNC-8, the specific capacity can still reach 750 mAh g^−1^ when the current density recovered to 0.5 A g^−1^ again. EIS spectra for samples SnO_2_@SNC-2 and SnO_2_@SNC-4 were also examined (Figure S7b), and the fitted resistance values are shown in Table S1. Among the three samples, sample SnO_2_@SNC-8 exhibits the simultaneously lowest solution resistance, charge transfer resistance and Warburg impedance. According to the previous reports, Rs values represent low internal resistance of the batteries, which is associated with the full penetration of the electrolyte into the active materials. Meanwhile, the decrease in the R_ct_ clearly indicate the quick charge transfer in the electrochemical reactions. According to the BET surface areas mentioned above, the surface areas of the samples increase with the increase of carbon contents. The surface area increase could effectively reduce the values of R_ct_ and R_s_ via enhancing the surface contact between the electrolyte and active materials, which is very important to maintain the cycling stability of the active materials.

## 4. Conclusions

In summary, SnO_2_@SNC composites were prepared by a simple and facile solid-state reaction. The as-prepared composite materials exhibit excellent cyclic stabilities owing to the introduction of S, N co-doped carbon. The experimental facts clearly indicate that the introduction of S, N co-doped carbon could effectively stabilize conversion or alloy reactions during the cycling process. By enhancing the reaction reversibility during the charge/discharge process, the long-life cycling performances of SnO_2_ can be greatly improved. EIS measurements clearly indicate the S and N co-doped carbon could improves the electric conductivity of the electrode, which may be the main reason for the improved reaction reversibility. Among the three samples, sample SnO_2_@SNC-8 exhibits the superior lithium storage performance. Even after cycling at 2 A g^−1^ for 1000 times, it can still deliver a discharge capacity of ~600 mAh g^−1^. By comparing the samples with different carbon contents, the BET surfaces areas are attributed to be the main reason for the differences in electrochemical performances. This simple and facile method not only provide a method for the synthesis of SnO_2_@SNC composite materials, but also shed new light on the optimization of SnO_2_ base electrode materials.

## Figures and Tables

**Table 1:** The fitted results of solution resistance (R_s_), the charge transfer resistance (R_ct_) and Warburg impedance (Z_w_) for SnO_2_@SNC−8 and SnO_2_ samples.

	SnO_2_@SNC−8	SnO_2_
R_ct_ (Ω)	166.9	391.5
R_s_ (Ω)	2.286	6.569
Z_w_ (Ω s^−1/2^)	127.3	320.2

## Data Availability

The data presented in this study are available on request from the corresponding author.

## Supplementary Materials

The following are available online at www.mdpi.com/article/10.3390/nano12040700/s1, Figure S1: (a) XRD pattern, (b) Raman and (c) TGA curve for the SnO_2_ sample; Figure S2: (a,b) SEM images, (c) TEM, (d) HRTEM and (e) SAED spectrum for the SnO_2_ sample. (f) HAADF image and elemental mapping for (g) Sn and (h) O element of pure SnO_2_; Figure S3: (a) XRD pattern, (b) TGA curve, (c) HRTEM, (d) HAADF image and elemental mapping for (e) Sn, (f) O and (g) C element for the SnO_2_@C sample; Figure S4: Long cyclic performance at 2 A g^-1^ for the SnO_2_@C composite; Figure S5: Galvanostatic charge/discharge voltage profiles at 100 mA g^−1^ in the first three cycles for the (a) SnO_2_@SNC−8 and (b) pure SnO_2_.; Figure S6: CV curves at a scan rate of 0.1 mV s^−1^ in the first three cycles for the (a) SnO_2_@SNC−2 and (b) SnO_2_@SNC−4; Figure S7: (a) Rate performance at different current density for SnO_2_@SNC samples. (b) EIS comparison for SnO_2_@SNC−2 and SnO_2_@SNC−4 and the corresponding equivalent circuit model; Table S1: The fitted results of solution resistance (R_s_), the charge transfer resistance (R_ct_) and Warburg impedance (Z_w_) for SnO_2_@SNC−8, SnO_2_@SNC−4 and SnO_2_@SNC−2 samples.
