# Supplementary material for: SnO2 Anchored in S and N Co-Doped Carbon as the Anode for Long-Life Lithium-Ion Batteries
Source: Nanomaterials (Basel). 2022 Feb 19;12(4):700. doi: 10.3390/nano12040700 (PMC8877561; doi:10.3390/nano12040700)
Supplement: Supplementary file 1 [file nanomaterials-12-00700-s001.zip › nanomaterials-1586558-supplementary.pdf]

nanomaterials

MDPI

Article

SnO2 Anchored in S and N Co-doped Carbon as an Anode for Long-life Lithium-Ion Batteries

Shuli Zhou [1], Hongyan Zhou [1], Yunpeng Zhang [1], Keke Zhu [1], Yanjun Zhai [1], Denghu Wei [1], Suyuan Zeng [1], *

[1] School of Chemistry and Chemical Engineering, Liaocheng University, 252059 Liaocheng, China; zhouli1996@126.com (S. Z.); zhy206533289@163.com (H.Z.); zyp2131@163.com (Y.Z.); zhukekexs@163.com (K.Z.); zhaiyanjun@lcu.edu.cn (Y.Z.); weidenghu@lcu.edu.cn (D.W.)

* Correspondence: drzengsy@163.com (S.Z.); Tel: +86-635-8230614, Fax: +86-635-8230196

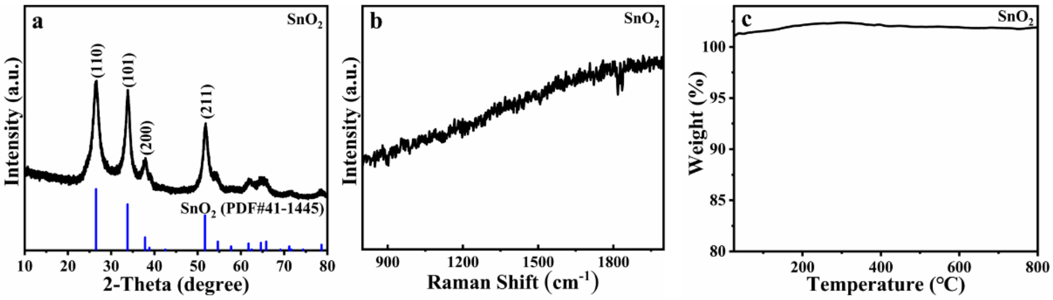

Figure S1. a) XRD pattern, **b)** Raman and **c)** TGA curve for the SnO_2 sample.

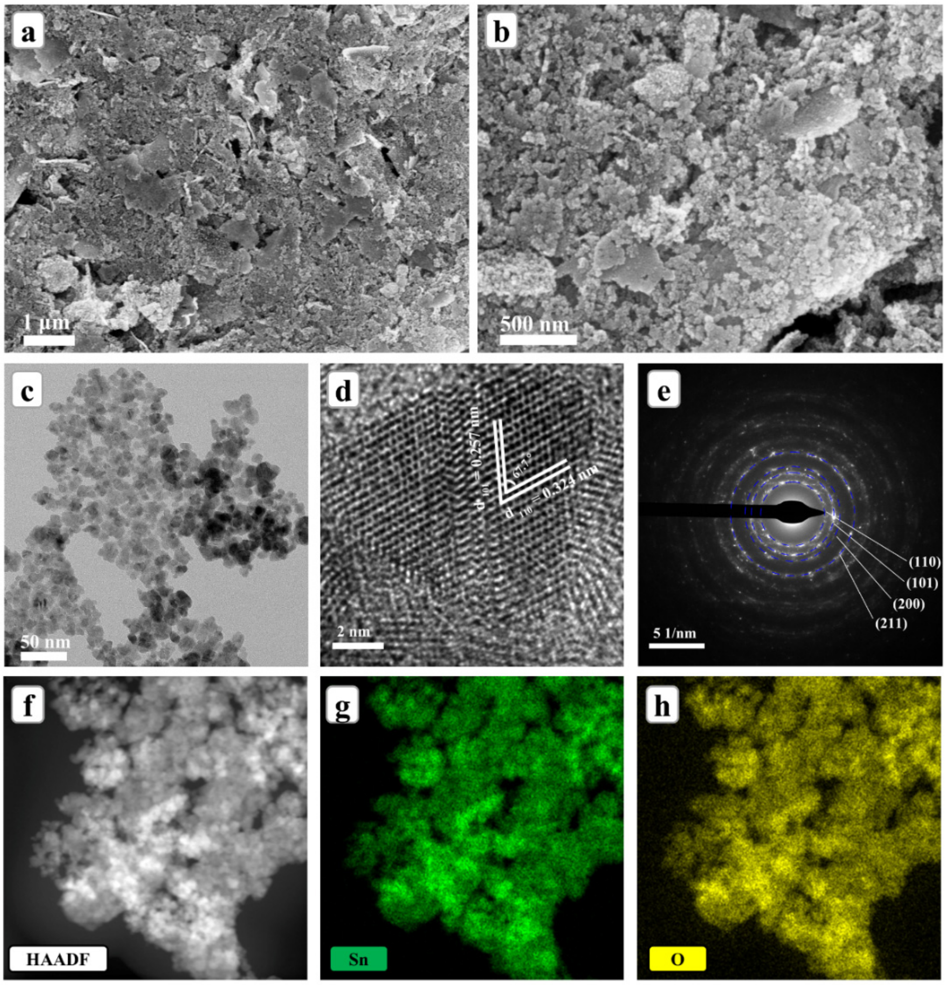

Figure S2. a-b) SEM images, **c)** TEM, **d)** HRTEM and **e)** SAED spectrum for the SnO_2 sample. **f)** HAADF image and elemental mapping for **g)** Sn and **h)** O element of pure SnO_2.

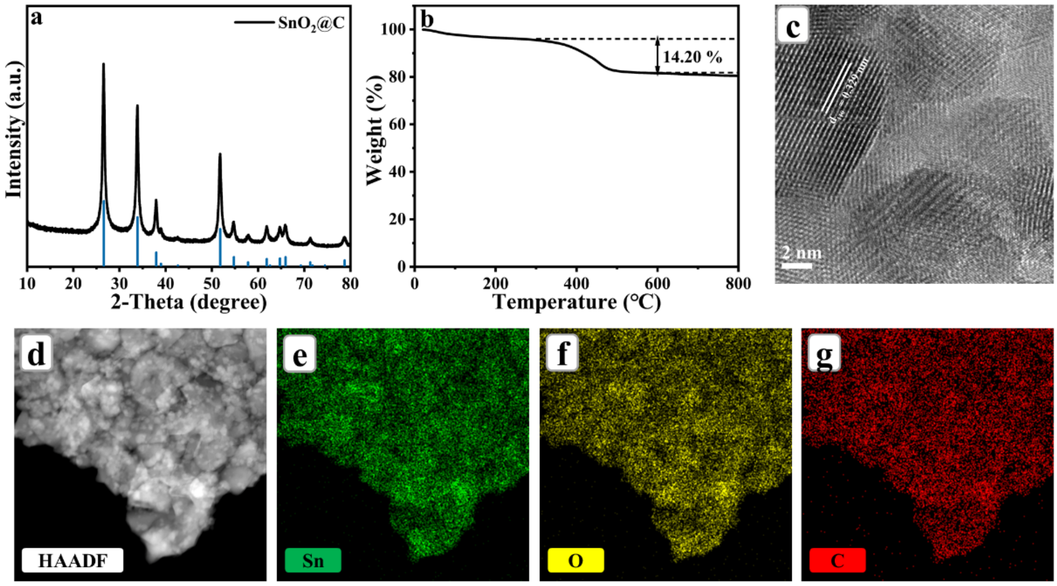

Figure S3. a) XRD pattern, **b)** TGA curve, **c)** HRTEM, **d)** HAADF image and elemental mapping for **e)** Sn, **f)** O and **g)** C element for the SnO_2@C sample.

Also, we prepared the SnO_2@C sample using starch as carbon source and the corresponding structure characterization was shown in Figure S3. Figure S3a shows the XRD patterns of the as-synthesized SnO_2@C materials without S/N co-doped, and all the diffraction peaks on which can be indexed to be a tetragonal phased SnO_2 (JCPDS Card No. 41-1445). To determine the carbon contents for the three SnO_2@SNC samples, thermogravimetric analysis (TGA) was employed and the carbon content of SnO_2@C materials was 14.20% (Figure S3b). The high-resolution transmission electron microscopy (HRTEM) image (Figure S3c) shows clear lattice fringe with an inter-planar spacing of 0.329 nm, which is consistent to the d-spacings of the (110) lattice planes of SnO_2. To further understand the elemental distribution of the SnO_2@C composite, the elemental mapping was carried out (Figure S3d-g). The experimental facts clearly show the well-distributed Sn, O and C elements.

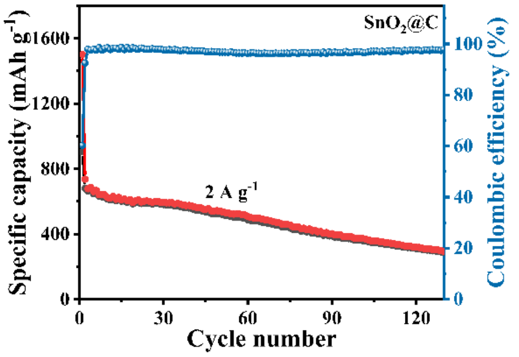

Figure S4. Long cyclic performance at 2 A g^{-1} for the SnO_2@C composite.

After cycling for 130 times under the current density of 2 A g^{-1}, the as-prepared sample SnO_2@C can deliver a discharge capacity of ~300 mAh g^{-1} (Figure S4).

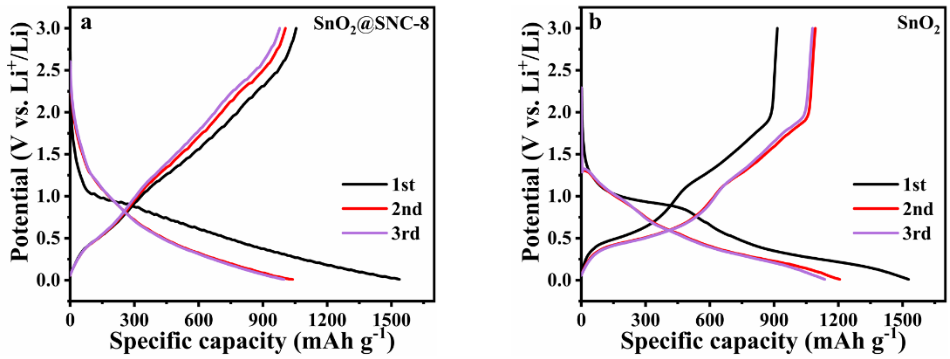

Figure S5. Galvanostatic charge/discharge voltage profiles at 100 mA g^{-1} in the first three cycles for the **a)** SnO_2@SNC-8 and **b)** pure SnO_2.

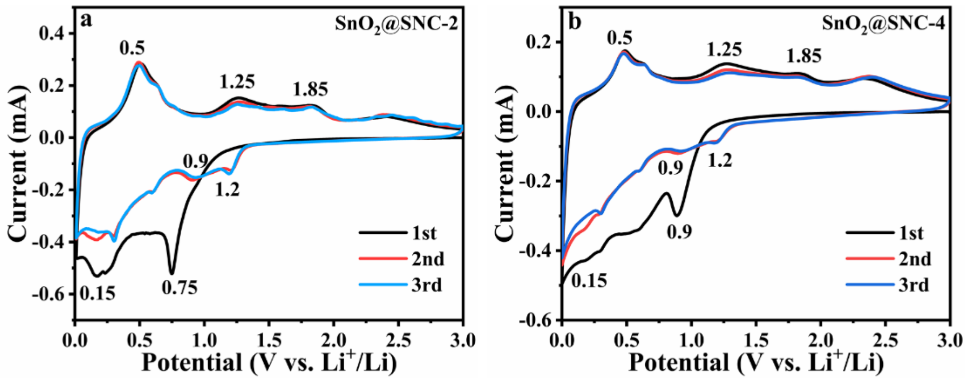

Figure S6. CV curves at a scan rate of 0.1 mV s^{-1} in the first three cycles for the **a)** SnO_2@SNC-2 and **b)** SnO_2@SNC-4.

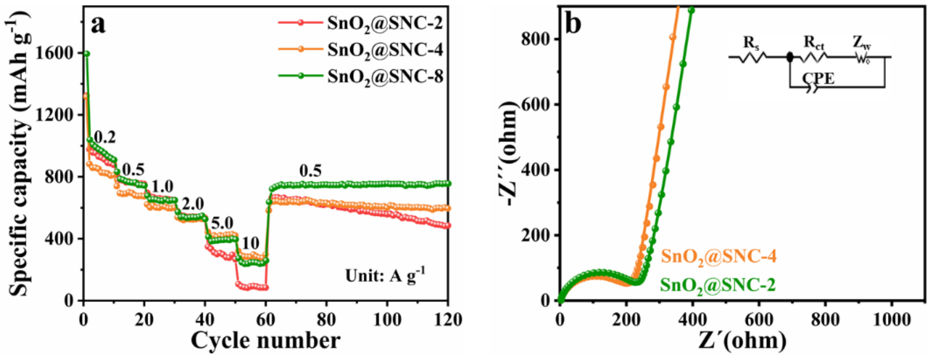

Figure S7. a) Rate performance at different current density for SnO_2@SNC samples. **b)** EIS comparison for SnO_2@SNC-2 and SnO_2@SNC-4 and the corresponding equivalent circuit model.

Table S1. The fitted results of solution resistance (R_s), the charge transfer resistance (R_{ct}) and Warburg impedance (Z_w) for SnO_2@SNC-8, SnO_2@SNC-4 and SnO_2@SNC-2 samples.

	**SnO_2@SNC-8**	**SnO_2@SNC-4**	**SnO_2@SNC-2**
R_{ct} (Ω)	166.9	166.2	192.8
R_s(Ω)	2.286	2.627	2.733
Z_w (Ω $s^{-1/2}$)	127.3	189.4	185.2
